# Complicated Clinical Course in Incipient Gigantism Due to Treatment-resistant Aryl Hydrocarbon Receptor–Interacting Protein–mutated Pediatric Somatotropinoma

**DOI:** 10.1016/j.aace.2021.12.003

**Published:** 2021-12-16

**Authors:** Selveta Sanne van Santen, Adrian F. Daly, Michael Buchfelder, Roland Coras, Yining Zhao, Albert Beckers, Aart Jan van der Lely, Leo J. Hofland, Rutger K. Balvers, P. van Koetsveld, Marry Marrigje van den Heuvel-Eibrink, Sebastian Johannes Cornelis Martinus Maria Neggers

**Affiliations:** 1 Department of Internal Medicine, Endocrinology; Erasmus Medical Center, Rotterdam, The Netherlands; 2 Princess Máxima Center for Pediatric Oncology, Utrecht, The Netherlands; 3 Department of Endocrinology, Liège University Hospital Centre, Liège University, Avenue de L’hopital, Liège, Belgium; 4 Department of Neurosurgery; University Hospital Erlangen, Erlangen, Germany; 5 Department of Neuropathology; University Hospital Erlangen, Erlangen, Germany; 6 Department of Neurosurgery; Erasmus Medical Center, Rotterdam, The Netherlands

**Keywords:** acromegaly, gigantism, *AIP* mutation, pituitary adenoma, macroadenoma, somatotropinoma, AIP, aryl hydrocarbon receptor–interacting protein, GH, growth hormone, IGF-I, insulin-like growth factor I, LAR, long-acting release, NR, normal range, SDS, standardised deviation scores, SSA, somatostatin analog, SSTR, somatostatin receptor

## Abstract

**Background:**

Our objective was to describe the clinical course and treatment challenges in a very young patient with a pituitary adenoma due to a novel *aryl hydrocarbon receptor–interacting protein (AIP)* gene mutation, highlighting the limitations of somatostatin receptor immunohistochemistry to predict clinical responses to somatostatin analogs in acromegaly.

**Case Report:**

We report the case of a 7-year-old boy presenting with headache, visual field defects, and accelerated growth following failure to thrive. The laboratory results showed high insulin-like growth factor I (IGF-I) (standardised deviation scores ( +3.49) and prolactin levels (0.5 nmol/L), and magnetic resonance imaging identified a pituitary macroadenoma. Tumoral/hormonal control could not be achieved despite 3 neurosurgical procedures, each time with apparent total resection or with lanreotide or pasireotide. IGF-I levels decreased with the GH receptor antagonist pegvisomant. The loss of somatostatin receptor 5 was observed between the second and third tumor resection. In vitro, no effect on tumoral GH release by pasireotide (with/without cabergoline) was observed. Genetic analysis revealed a novel germline *AIP* mutation: p.Tyr202∗ (pathogenic; class 4).

**Discussion:**

In vitro response of tumor tissue to somatostatin may better predict tumoral in vivo responses of somatostatin analogs than somatostatin receptor immunohistochemistry.

**Conclusion:**

We identified a novel pathologic *AIP* mutation that was associated with incipient acrogigantism in an extremely young patient who had a complicated course of disease. Growth acceleration can be masked due to failure to thrive. Tumoral growth hormone release in vivo may be predicted with in vitro exposure to somatostatin receptor analogs, as it cannot be assumed that all *AIP*-mutated somatotropinomas respond well to pasireotide.

## Introduction

Pituitary adenomas have a prevalence of 1 clinically-relevant case per 1000 adults.^1^ Most pituitary adenomas are sporadic, but 5% have a familial background, the most common being familial isolated pituitary adenomas.^1^^,^^2^ In familial isolated pituitary adenomas, 15% to 30% of cases are associated with pathologic germline variants in the *aryl hydrocarbon receptor–interacting protein* (*AIP*) gene, a tumor suppressor gene located on chromosome 11q13.^2^, ^3^, ^4^, ^5^, ^6^ Germline *AIP* mutations are particularly associated with growth hormone- (GH) or mixed GH-prolactin–secreting pituitary adenomas.^3^, ^4^, ^5^, ^6^ Patients with *AIP* mutations are often men and have an aggressive clinical phenotype due to large invasive tumors. *AIP* mutations are the most frequent genetic cause of pituitary gigantism (29%).^2^^,^^5^^,^^7^^,^^8^ In large case series, *AIP-*mutated pituitary adenomas usually present in adolescence or early adulthood.^9^ Early pediatric presentations of patients with *AIP* mutations and GH-secreting pituitary adenomas are rarely described, and responses to medical and surgical management in this challenging population are not well-understood. Here, we report the challenges faced in the presentation, diagnosis, and management of a young boy with a novel *AIP* mutation that led to a recurrent and resistant GH-secreting macroadenoma.

## Case Description

**Fig. 1:**
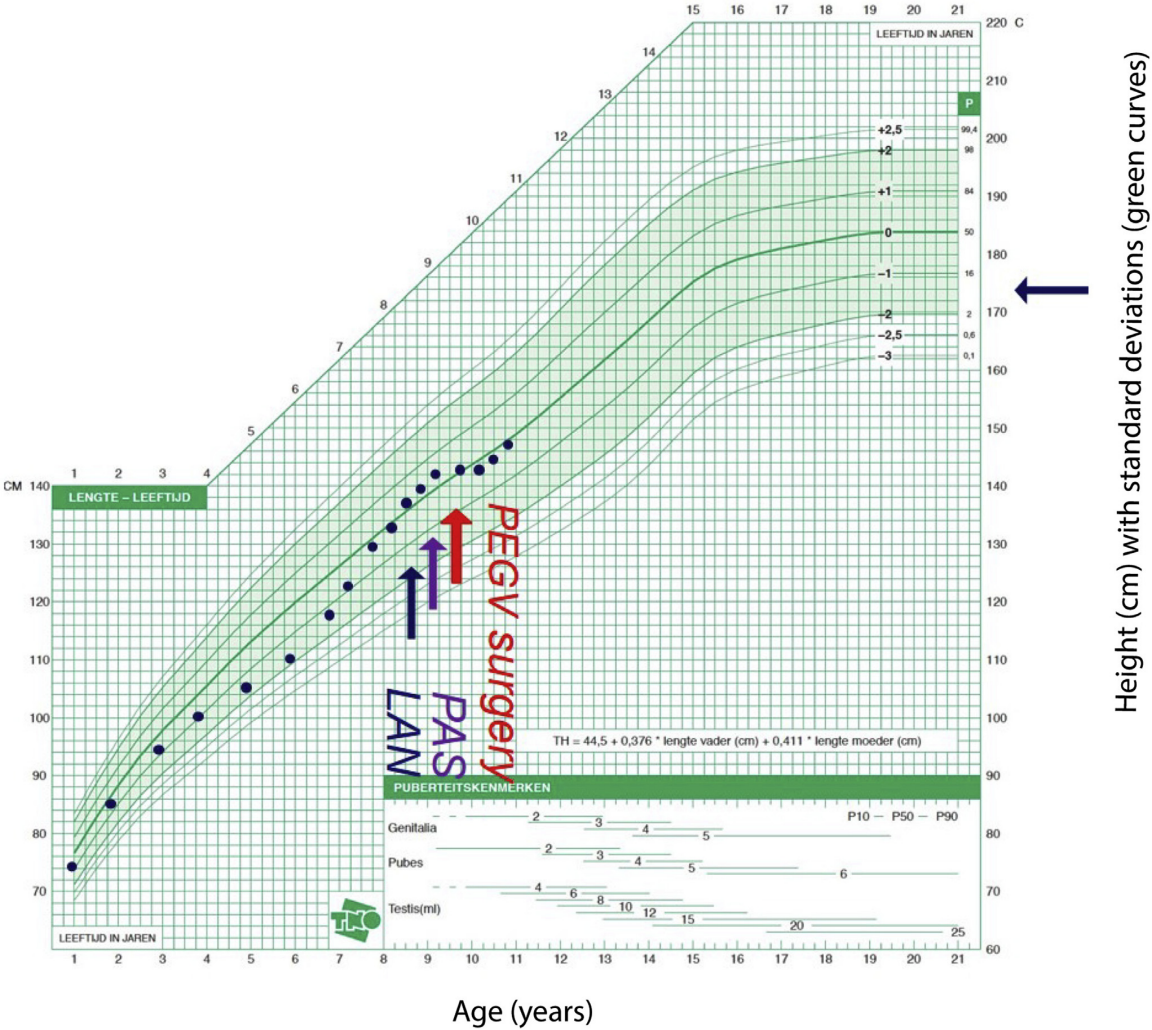
Growth chart of the patient with incipient gigantism. The initial normal growth of the patient declined from 3 to 6 years of age, but then deflected markedly upward. The patient was diagnosed at the age of 7 years. The blue arrow corresponds with LAN treatment, the purple arrow corresponds with PAS treatment, and the red arrow corresponds with PEGV surgery. One month after switching to pasireotide, the first transsphenoidal resection was performed. Two months thereafter, pegvisomant was started. After 1 month of pegvisomant, the tumor volume increased; pegvisomant was stopped, and a second resection followed. Six months thereafter, the third surgery was performed. *LAN* = lanreotide; *PAS* = pasireotide; *PEGV surgery* = pegvisomant and surgery.

**Fig. 2:**
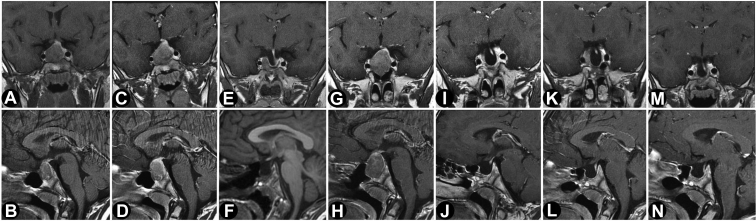
Sequential magnetic resonance imaging over the clinical course of the patient between 2018 and 2020. Contrast-enhanced T1-weighted sequences in coronal (*A, C, E, G, I, K, M*) and sagittal (*B, D, F, H, J, L, N*) planes were chosen and corrected for gray scale and magnification. The timing of the scans were as follows: at clinical presentation (*A, B*); before the first operation (lanreotide was switched to pasireotide; after this, magnetic resonance imaging was performed because of tumor growth, and surgery was performed because of visual field defects due to chiasmic compression) (*C, D*); postoperatively after the first operation (*E, F*); before the second operation (*G, H*); postoperatively after the second operation (1 month after intiating pegvisomant treatment) (*I, J*); before the third operation (*K, L*); and postoperatively after the third operation (*M, N*). There was no inhibition of tumor growth after the use of somatostatin analogs in terms of tumor size and extent. Correspondingly, the growth hormone secretion was normalized after the respective tumor resections. The tumor was medial to the intracavernous intercarotid line (Knosp status grade II); however, on direct vision during the last surgery, there was invasion of the cavernous sinus wall.

**Fig. 3:**
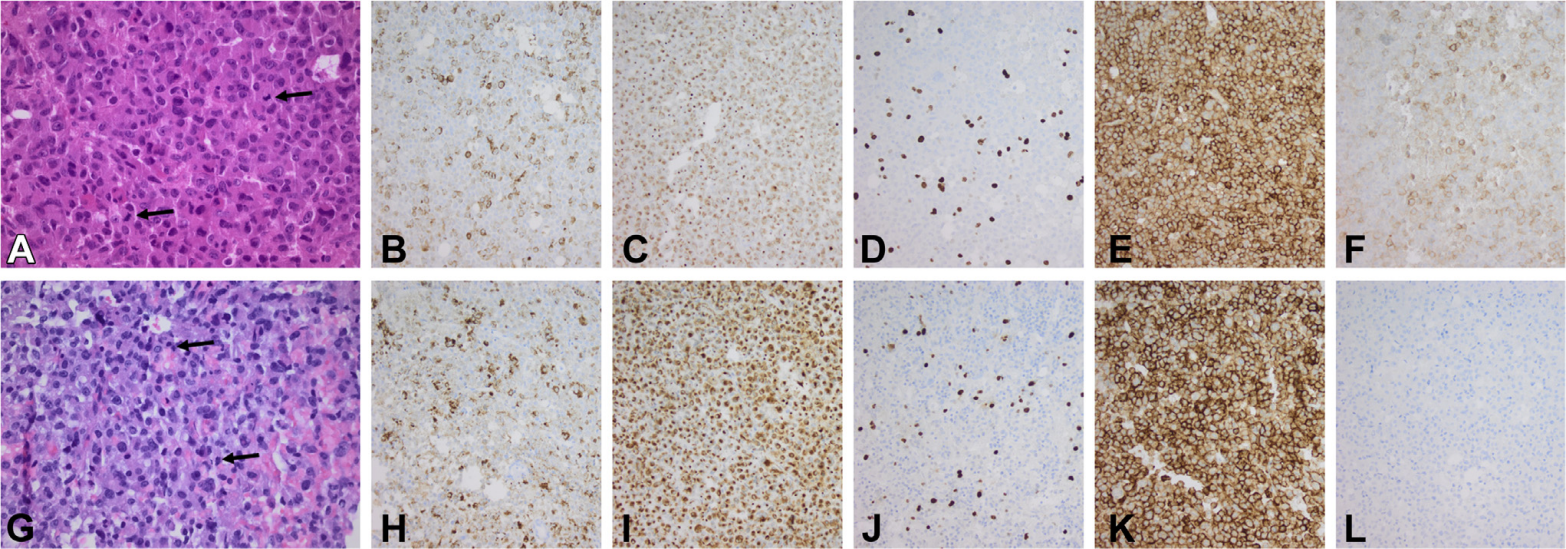
Histopathologic features of the tumor at the second and third surgery. The tissue from the first surgery was unavailable. *A-F*, the second surgery; *G-L*, the third surgery. *A* and *G*, Hematoxylin and eosin staining show a pituitary adenoma with interspersed mitoses in both surgeries (black arrows). *B and H*, Growth hormone expression. *C* and *I*, PanCK immunohistochemistry shows a few fibrous bodies in both specimens. In both specimens, there is an increased proliferation activity (Ki67 staining in *D* and *J*). The tissue from both the surgeries had a homogeneous expression of SSTR2 (*E, K*), whereas SSTR5 was moderately expressed in the specimen of the second surgery (*F*) and absent in the tissue of the third surgery (*L*).

A 7-year-old boy was hospitalized for the evaluation of multiple progressive complaints over the previous 2 years, including frontal headache, fatigue, tics, leg pain, nocturnal sweating, constipation, and poor food intake. He had a normal birthweight/height following an unremarkable pregnancy, and his family history was normal. His growth curve showed normal growth until the age of 3 years, followed by a marked decrease to about −2 standardised deviation scores (SDS) at the age of 6 years (Fig. 1). Thereafter, his growth increased rapidly compared with the Dutch national standards. His parents were of modest stature (father, 170 cm and mother, 164 cm) by the current Dutch median height standards (men, 182.9 cm and women, 169.3 cm). A sellar tumor with an enlarged sella turcica was discovered (Fig. 2). Laboratory analysis showed an insulin-like growth factor I (IGF-I) level of 56.5 nmol/L (normal range [NR], 8.3-38.2; SDS, +3.49), prolactin level of 0.5 nmol/L (NR, <0.36 nmol/L), TSH level of 1.07 mU/L (NR, 0.6-5.6 mU/L), free thyroxine level of 20.2 pmol/L (NR, 13-26 nmol/L), afternoon cortisol level of 270 nmol/L (NR, <700 nmol/L), and undetectable luteinizing hormone and follicle-stimulating hormone (normal for prepubertal state).^10^ Over time, the growth rate accelerated further (Fig. 1), in parallel with rising IGF-I (71.2 nmol/L; +4.53 SDS), a random GH of 30.8 μg/L (NR, <4.0 μg/L), and prolactin increase to 0.75 nmol/L. The nadir GH value during an oral glucose tolerance test was 26.7 μg/L. He complained of vomiting and loss of appetite. Treatment of the GH-secreting macroadenoma was initiated with lanreotide 120 mg once in 4 weeks, which resulted in no biochemical response (IGF-I level, 76.6 nmol/L and GH level, 28.0 μg/L) or inhibition of tumor growth after 4 doses. Lanreotide was switched to pasireotide 60-mg long-acting release (LAR) once in 4 weeks. One month after switching, he developed a new onset of bitemporal field defects and headaches that indicated symptomatic optic chiasmal compression, and he underwent transsphenoidal surgery for the first time. Two months postoperatively (3 months after the initiation of pasireotide LAR), IGF-I (70.3 nmol/L) and GH (23.4 μg/L) levels remained elevated. Pasireotide LAR showed no hormonal or tumoral effects, and the GH receptor antagonist pegvisomant was started with a weekly dose of 40 mg. Although IGF-I levels dropped to 34.1 nmol/L (NR, 10.9-47.3 nmol/L; 0.87 SDS), the local GH assay, which does not detect pegvisomant, continued to show an elevated random GH level (48.6 μg/L). After 1 month of pegvisomant, severe headaches returned, and bitemporal hemianopsia reoccurred due to an increase in tumor volume (Fig. 2). Pegvisomant was stopped, and a second transsphenoidal resection followed (Fig. 2). The histopathologic report revealed a pituitary adenoma staining positive for GH and negative for prolactin (Fig. 3). One month after the second transsphenoidal surgery, his IGF-I level declined to 29.3 nmol/L (0.4 SDS), GH level was 2.7 μg/L, and prolactin level declined from 0.60 to 0.38 nmol/L (NR, 0.1-0.5 nmol/L). Five months after surgery, the headaches returned, and magnetic resonance imaging 1 month later showed a small remnant lateral to the right internal carotid artery (Fig. 2). IGF-I increased again to +2.9 SDS. A third transsphenoidal surgery was performed, leading to the normalization of GH and IGF-I levels. Thirteen months after his last surgery, he received stereotactic radiotherapy (54 Gy), and 4 months after radiotherapy, his last IGF-I was −0.8 SDS.

Due to the presentation with a macroadenoma at a young age, germline genetic testing for sequence variants and deletions in *AIP* and *MEN1* genes was performed. A novel heterozygotic truncating variant in the *AIP* gene was discovered (c.606C>G; p.Tyr202∗; GnomAD database mean allele frequency (0), which was accompanied by a second missense variant (c.695C>T: p.Pro232Leu; mean allele frequency, 0.00002502), both of which were paternally inherited. Screening by magnetic resonance imaging and hormone evaluation of his 37-year-old father was negative.

**Fig. 4:**
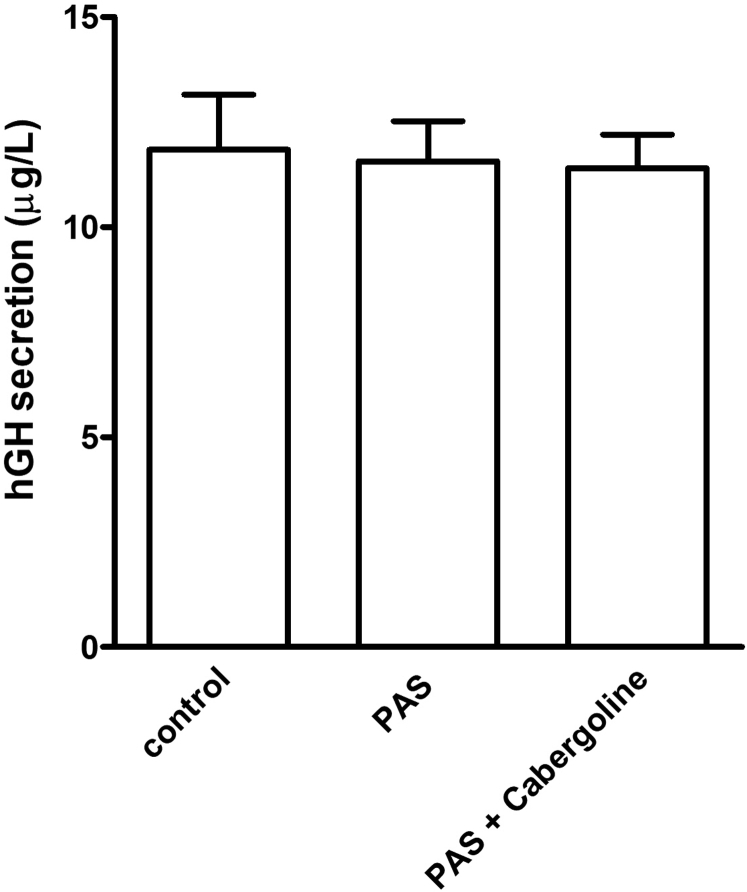
In vitro sensitivity of the cultured tumor cells to pasireotide and cabergoline. Growth hormone secretion by primary cultured adenoma cells of the patient did not respond to incubation with pasireotide (10 nM) or pasireotide (10 nM) plus cabergoline (10 nM); there was no statistically significant change in the growth hormone secretion after a 72-hour incubation with the drugs. The cells were cultured as a monolayer in a 250-μL medium in a 48-well culture plate. The tumor cell isolation and culture conditions were as described by Hofland et al.^17^ The medium growth hormone concentrations are expressed in μg/L and are mean ± SD (n = 3 wells per group). Data were analyzed by a 1-way analysis of variance, and multiple comparisons between groups were assessed with the Newman-Keuls test using GraphPad Prism. *hGH* = human growth hormone; *PAS* = pasireotide.

Histopathologic analysis revealed a loss of SSTR5 expression between the second and third operations (Fig. 3). In vitro characterization of the second surgical sample showed no statistically significant inhibition of GH secretion to incubation with pasireotide (10 nM) or coincubation with pasireotide and cabergoline (both 10 nM; Fig. 4). Other compounds could not be tested due to the limited amount of available tissue. These interesting findings should be confirmed in a wider series of tumors from patients with *AIP* mutations and in appropriate wild-type acromegaly controls.

Given the lack of tumor size control with first- and second-generation somatostatin analogs (SSAs) and the unresectable remnant that required radiotherapy at the age of 10 years, the patient will require intensive (endocrinological) follow-up, although no pituitary deficiencies have occurred to date. If needed, excessive GH can be controlled by pegvisomant, albeit with high vigilance for tumor regrowth.

## Discussion

This case report describes a complicated somatotropinoma leading to accelerated longitudinal growth that was masked by an unexpected initial period of failure to thrive, which likely occurred due to poor feeding because of nausea. The disease was diagnosed at the very young age of 7 years and was found to be due to a previously undescribed *AIP* mutation that was inherited from his unaffected father. Decreased clinical SSA sensitivity may be related to the evolving tumor biology between surgeries, particularly the loss of tumoral somatostatin receptor (SSTR) 5 expression, whereas in vitro, there was no tumoral response of GH to pasireotide and cabergoline.

Somatotropinomas are primarily treated with (transsphenoidal) neurosurgery, SSAs, dopamine agonists, or GH receptor antagonists.^11^ Overall, in acromegaly, long-acting SSAs can achieve biochemical normalization of GH and IGF-I in 50% to 60% and often lead to modest tumor shrinkage.^11^, ^12^, ^13^, ^14^ Patients with *AIP* mutations have, however, significantly less tumor shrinkage and lower hormonal responses to first-generation SSAs.^7^

SSAs act via SSTRs1 to 5.^6^ The first-generation SSAs octreotide and lanreotide have the highest affinity for SSTR2 and have a low affinity to SSTR3 and SSTR5, whereas the second-generation SSA pasireotide has the highest affinity for SSTR5, followed by SSTR2, SSTR3, and SSTR1.^6^^,^^15^ As reported previously, pasireotide resistance is possibly more related to SSTR2 expression than to SSTR5 in the general acromegaly population.^16^^,^^17^ SSA resistance may occur if the tumor is lacking SSTR2.^6^ Daly et al^18^ recently reported 2 *AIP*-mutated acromegaly patients with resistance to first-generation SSA, in whom pasireotide treatment led to marked tumor shrinkage and persistent hormonal control. In 1 case, very low-to-absent SSTR2 levels were seen, and the efficacy of pasireotide must have been through other SSTRs like SSTR5.^18^ Due to this, we initially expected that our patient would respond better to pasireotide despite resistance to first-generation SSAs, but this was not the case.^18^ The resistance to pasireotide probably relates in part to the low SSTR5 expression, since SSTR2 expression remained present. Nevertheless, signaling via SSTR2 may be affected while leaving the receptor expression unaffected. Possible factors in this phenomenon include ZAC1 and miR-34a, both of which influence SSTR2 signaling.^11^^,^^19^ In these cases, it may be preferable to test the in vitro response of the tumor tissue assessed by decreases in GH secretion (17). In the study by Coopmans et al^20^ that included 45 acromegaly patients who were previously treated with first-generation SSAs combined with pegvisomant, SSTR2 immunoreactivity scores were found to be related to significant tumor shrinkage in patients treated with pasireotide, which was not the case for SSTR5. Muhammad et al^16^ found in the same cohort that IGF-1 lowering effects of pasireotide correlated with SSTR2 instead of SSTR5. However, the timing of the change in responsiveness and change in SSTR5 expression occurred simultaneously in the current case. In the study by Iacovazzo et al^21^ that included 39 patients with somatotropinomas, SSTR5 expression predicted responsiveness to pasireotide.

Our case exemplifies the many challenges that can be faced in the recognition of acromegaly, especially when occurring at an extremely young age. Acrogigantism can occur with increased growth velocity in young patients, even without extremely elevated height compared to age-/sex-matched references. An appreciation of the totality of the abnormal growth characteristics is important when assessing children with aberrant growth. In this case, a novel *AIP* mutation, p.Tyr202∗, was found. The unresponsiveness of the tumor to pasireotide may be better assessed by in vitro responsiveness as opposed to somatostatin receptor evaluation. Future studies are necessary to test this hypothesis in cohorts with more patients and with a control group.

## Conclusion

This informative case of incipient gigantism in a 7-year-old child with a novel *AIP* mutation, p.Tyr202∗, was associated with a highly treatment-resistant somatotropinoma. Although previous literature suggests a favorable response to pasireotide in some patients with *AIP* mutations and acromegaly, pasireotide had only limited effect in our patient, possibly related to decreasing SSTR5 expression of the tumor.^18^ In vitro GH suppression in the cultured tumor tissue may predict in vivo treatment response better than SSTR assessment. Genetic testing of the *AIP* gene should be advocated in all patients with GH-secreting pituitary adenomas occurring in childhood and/or (incipient) pituitary gigantism.^22^

## Disclosure

The authors have no multiplicity of interest to disclose.
